# Compatibility of pedigree-based and marker-based relationship matrices for single-step genetic evaluation

**DOI:** 10.1186/1297-9686-44-37

**Published:** 2012-12-03

**Authors:** Ole F Christensen

**Affiliations:** 1 Center for Quantitative Genetics and Genomics, Department of Molecular Biology and Genetics, Aarhus University, Blichers Allé 20, P.O. BOX 50, DK-8830 Tjele, Denmark

## Abstract

**Background:**

Single-step methods provide a coherent and conceptually simple approach to incorporate genomic information into genetic evaluations. An issue with single-step methods is compatibility between the marker-based relationship matrix for genotyped animals and the pedigree-based relationship matrix. Therefore, it is necessary to adjust the marker-based relationship matrix to the pedigree-based relationship matrix. Moreover, with data from routine evaluations, this adjustment should in principle be based on both observed marker genotypes and observed phenotypes, but until now this has been overlooked. In this paper, I propose a new method to address this issue by 1) adjusting the pedigree-based relationship matrix to be compatible with the marker-based relationship matrix instead of the reverse and 2) extending the single-step genetic evaluation using a joint likelihood of observed phenotypes and observed marker genotypes. The performance of this method is then evaluated using two simulated datasets.

**Results:**

The method derived here is a single-step method in which the marker-based relationship matrix is constructed assuming all allele frequencies equal to 0.5 and the pedigree-based relationship matrix is constructed using the unusual assumption that animals in the base population are related and inbred with a relationship coefficient *γ* and an inbreeding coefficient *γ* / 2. Taken together, this *γ* parameter and a parameter that scales the marker-based relationship matrix can handle the issue of compatibility between marker-based and pedigree-based relationship matrices. The full log-likelihood function used for parameter inference contains two terms. The first term is the REML-log-likelihood for the phenotypes conditional on the observed marker genotypes, whereas the second term is the log-likelihood for the observed marker genotypes. Analyses of the two simulated datasets with this new method showed that 1) the parameters involved in adjusting marker-based and pedigree-based relationship matrices can depend on both observed phenotypes and observed marker genotypes and 2) a strong association between these two parameters exists. Finally, this method performed at least as well as a method based on adjusting the marker-based relationship matrix.

**Conclusions:**

Using the full log-likelihood and adjusting the pedigree-based relationship matrix to be compatible with the marker-based relationship matrix provides a new and interesting approach to handle the issue of compatibility between the two matrices in single-step genetic evaluation.

## Introduction

Single-step methods for genetic evaluation [1-3] have recently become popular because they provide an approach to incorporate genomic information into genetic evaluations that is both coherent and conceptually simple. A single-step method extends the usual pedigree-based method by replacing the additive relationship matrix constructed from pedigree by an additive relationship matrix that combines the marker-based relationship matrix for genotyped animals with the pedigree-based relationship matrix.

An issue with a single-step method is compatibility between the marker-based relationship matrix for genotyped animals and the pedigree-based relationship matrix [4-6]. To handle this problem, it is necessary to determine which allele frequencies should be used in the marker-based relationship matrix and to adjust this matrix to the pedigree-based relationship matrix. In theory, one should use the allele frequencies in the founder population of the pedigree (base animals) for the marker-based and pedigree-based relationships to be compatible, but these allele frequencies are rarely available in practice since base animals are not genotyped. Chen et al. [5] and Forni et al. [7] concluded that using the observed allele frequencies improved accuracy of prediction compared to using allele frequencies equal to 0.5. Studies on how to adjust the marker-based relationship matrix to be compatible with the submatrix of the pedigree-based relationship matrix for genotyped animals have been reported [4-6]. These adjustments consisted of scaling and adding a number to all elements in the marker-based relationship matrix based on equating means of diagonal and off-diagonal elements in the two matrices, and it was demonstrated that accuracy of prediction increased and bias decreased. In relation to this problem of compatibility between marker-based and pedigree-based relationship matrices, with data from routine evaluations, selection affects the allele frequencies over time, and in principle both observed marker genotypes and observed phenotypes contain information about allele frequencies in the base population. Therefore, studies on the adjustment of the marker-based relationship matrix to the pedigree-based relationship matrix have overlooked the fact that it should in principle incorporate information on observed phenotypes.

This work explores two possibilities to solve the problem, i.e. 1) in which the pedigree-based relationship matrix is adjusted to the marker-based relationship matrix and 2) in which the single-step genetic evaluation is extended by using a joint likelihood of observed phenotypes and observed marker genotypes. This results in a single-step method in which the marker-based relationship matrix is constructed assuming all allele frequencies are equal to 0.5 and the pedigree-based relationship matrix is constructed using the unusual assumption that animals in the base population are related and inbreed with a relationship coefficient *γ* and an inbreeding coefficient *γ* / 2. The log-likelihood function used for parameter inference contains two terms. The first term is the REML-log-likelihood for the phenotypes conditional on the observed marker genotypes and the second term is the log-likelihood for the observed marker genotypes. The performance of the proposed method was evaluated using two simulated datasets.

## Methods

This section presents, first, the statistical model on which the single-step methods are based along the lines of Christensen and Lund [3], then the proposal on how to adjust the pedigree-based relationship matrix and finally the adjustment of the marker-based relationship matrix as previously used.

### Single-step model

The marker genotypes are summarised into a marker matrix **m**, where *m*_*ij*_ = −1, 0 or 1 if SNP *j* of individual *i* is 11, 12, or 22, respectively. In the following, capital **M** and lowercase **m** indicate whether marker genotypes are considered as random variables or as non-random variables (observed variables or integration variables), respectively.

Let us consider the simple model

y=μ1+Za+e,

where **y** is the vector of phenotypes, *μ* is the general mean, **1** is a vector of ones, **a** is the vector of breeding values, **Z** is an incidence matrix, and **e** is the vector of residual errors. The breeding value may be decomposed into **a** = **g** + **a**_*r*_, where **g** is the vector of genomic effects and **a**_*r*_ = (**a** − **g**) is the vector of residual polygenic effects. The residual polygenic effects $a_{r}∼N(0,\sigma_{a}^{2}\omega A)$, where matrix **A** is the pedigree-based additive relationship matrix and *ω* ∈[0;1] is the relative weight on the residual polygenic effect. The genomic values $\left[g|M]∼N(0,\sigma_{a}^{2}(1-\omega )G(M)\right)$, with $\left(G\left(M\right)=\left(M-\left(2\rho -1\right)1^{T}\right){\left(M-\left(2\rho -1\right)1^{T}\right)}^{T}/s\right)$, where ***ρ*** = (*ρ*_1_, … , *ρ*_*p*_) with *ρ*_*j*_ being the allele frequency for the *j*th marker and $s=s(v)=\underset{j}{\sum }v_{j}$ with *v*_*j*_ being defined below. The model for the marker genotypes is that **M** is a multivariate Gaussian distribution with

$$\text{E}[M_{\text{ij}}|\rho_{j}]=2\rho_{j}-1,\;\text{Cov}[M_{\text{ij}},M_{i^{\prime }j}|v_{j}]=v_{j}A_{ii^{\prime }},$$

$$\text{Cov}[M_{\text{ij}},M_{i^{\prime }j^{\prime }}|v_{jj^{\prime }}]=v_{jj^{\prime }}A_{ii^{\prime }},$$

where *v*_*j*_ = *v*_*j*,*j*_ is a parameter and $v_{jj^{\prime }}=0$ for *j* ≠ *j*^*′*^. The crude assumption that the multivariate distribution is Gaussian is crucial in the derivation, whereas the unrealistic assumption that $v_{jj^{\prime }}=0$ for *j* ≠ *j*^*′*^ is made for simplicity. Dividing marker genotypes **m** into observed marker genotypes **m**^*o*^ and un-observed marker genotypes **m**^*u*^, the joint marginal density of observed phenotypes **y** and observed marker genotypes **m**^*o*^ is

$$\begin{array}{lll} & f(y,m^{o})=\int f(y,m^{o},m^{u})dm^{u}\; & \;\\ & =\int f(y|m^{o},m^{u})f(m^{u}|m^{o})f(m^{o})dm^{u},\; & \; \end{array}$$

where

$$\begin{array}{lll} & f(y|m^{o},m^{u})\; & \;\\ & =\int \int \int f(y|a_{r},g,\mu )f(a_{r})f(g|m^{o},m^{u})da_{r}dg\mathrm{d\mu .}\; & \; \end{array}$$

By rearranging terms and using that $\int f(g|m^{o},m^{u})f(m^{u}|m^{o})dm^{u}=f(g|m^{o})$, this becomes

$$\begin{array}{lll} & f(y,m^{o})\; & \;\\ & =\int \int \int f(y|a_{r},g,\mu )f(a_{r})f(g|m^{o})da_{r}dg\mathrm{d\mu}\; & \;\\ & \;\times f(m^{o})\; & \;\\ & =f(y|m^{o})f(m^{o}),\; & \; \end{array}$$

where *f*(**y**∣**m**^*o*^) is defined implicitly. The first term *f*(**y**∣**m**^*o*^) is the density for the phenotypes given the observed marker genotypes, whereas the second term *f*(**m**^*o*^) is the multivariate Gaussian density for the observed marker genotypes.

Single-step methods are based on the density *f*(**y**∣**m**^*o*^), which may be written (see [3]) as

$$f(y|m^{o})=\int \int f(y|a,\mu )f(a|m^{o})da\mathrm{d\mu},$$

where the vector **a** has a mean zero and a variance-covariance matrix **H**_*ω*_ with inverse

(1) $$H_{\omega }^{-1}=\left[\begin{array}{cc} G_{\omega }^{-1}-A_{11}^{-1} & 0\\ \;0 & 0 \end{array}\right]+A^{-1},$$

with **G**_*ω*_ = (1 − *ω*)**G**(**m**^*o*^) + *ω***A**_11_. In the above formula, the sub-division is made according to genotyped and non-genotyped animals. The matrices $\left(G\left(m^{o}\right)=\left(m^{o}\;-\;\left(2\rho \;-\;1\right)1^{T}\right){\left(m^{o}\;-\;\left(2\rho \;-\;1\right)1^{T}\right)}^{T}/s\right)$ and **A**_11_ are the marker-based relationship matrix for the genotyped animals and the submatrix of the pedigree-based relationship matrix corresponding to genotyped animals, respectively. The sparse structure of the matrix in equation (1) is the corner-stone for efficient computing using a single-step method. Assuming that **a**∣**m**^*o*^ is Gaussian distributed, mixed model equations can be solved for BLUP predictions and AI-REML [8] provides REML parameter estimates.

Issues raised by the single-step method are 1) how are the allele frequencies ***ρ*** that are used in **G**(**m**^*o*^) obtained and 2) how is the required compatibility between **G**(**m**^*o*^) and **A**_11_ that is evident in equation (1) reached. To investigate these issues, the joint density of observed phenotypes and marker genotypes, *f*(**y**, **m**^*o*^) = *f*(**y**∣**m**^*o*^)*f*(**m**^*o*^), is taken as the starting point. From this, the full marginal log-likelihood becomes

(2) $$\begin{array}{lll} & \ell (\sigma_{a}^{2},\sigma_{e}^{2},\omega ,\rho ,v)\; & \;\\ & =\ell_{y|m^{o}}(\sigma_{a}^{2},\sigma_{e}^{2},\omega ,\rho ,s(v))+\ell_{\text{mark}}(\rho ,v),\; \end{array}$$

where $\ell_{y|m^{o}}(\sigma_{a}^{2},\sigma_{e}^{2},\omega ,\rho ,s(v))$ for fixed *ω*, ***ρ*** and $s(v)=\underset{j}{\sum }v_{j}$ is the single-step REML-log-likelihood for the phenotypes conditional on the observed marker genotypes, and

$$\begin{array}{lll} & \ell_{\text{mark}}(\rho ,v)=\text{const}-(n_{1}/2)\underset{j}{\sum }\text{log}(v_{j})\; & \;\\ & -\underset{ii^{\prime }j}{\sum }\frac{\left(m_{\mathrm{ij}}^{o}-\left(2\rho_{j}-1\right)){\left(A_{11}^{-1}\right)}_{ii^{\prime }}(m_{i^{\prime }j}^{o}-\left(2\rho_{j}-1\right)\right)}{2v_{j}},\; & \; \end{array}$$

with *n*_1_ denoting the number of genotyped animals, is the log-likelihood of the observed marker genotypes. Thus, the parameter ***ρ*** enters into both terms of the full log-likelihood in equation (2), and this implies that estimation of allele frequencies ***ρ*** should be in principle based on this log-likelihood. In particular, if selection has been performed the phenotypes will contain information about the allele frequencies which may cause bias if ignored. However, estimating ***ρ*** and *s* = *s*(**v**) by maximising $\left(\ell (\sigma_{a}^{2},\omega ,\sigma_{e}^{2},\rho ,v)\right)$ for all parameters jointly is not feasible in practice since ***ρ*** is a very high-dimensional parameter. Instead, in general the observed marker genotypes are used to estimate ***ρ*** and then they are plugged into $\left(\ell_{y|m^{o}}\right)$, and this log-likelihood is used to estimate the remaining parameters. Estimation of ***ρ*** based on observed marker genotypes may consist of simply using the observed allele frequencies [5,7], or may be done by maximising *ℓ*_*mark*_(***ρ*v**) as a function of ***ρ*** (this is essentially the method of Gengler et al. [9], although, for computational reasons, that method adds a small residual error to the distribution of **M**). The high-dimensional parameter **v** also enters into both terms of the full log-likelihood, although it only enters into $\left(\ell_{y|m^{o}}\right)$ via the scaling parameter *s* = *s*(**v**), and therefore estimation of **v** should also be in principle based on maximising the full log-likelihood. Usually, estimating $s=\underset{j}{\sum }v_{j}$ is based on the observed marker genotypes, either using $v_{j}=2{\hat{\rho }}_{j}(1-{\hat{\rho }}_{j})$, implying $s=\underset{j}{\sum }2{\hat{\rho }}_{j}(1-{\hat{\rho }}_{j})$ as in [2,3], or using *s* such that the average diagonal of **G** equals the average diagonal of **A**_11_ as in [7]. Furthermore, it has been demonstrated that the accuracy of prediction is improved and bias is reduced by adjusting the marker-based relationship matrix as **G**(**m**^*o*^)*β* + *α* where *α* and *β* are based on the elements in **G**(**m**^*o*^) and **A**_11_; further details are given below. It should be noted that such an adjustment is based on observed marker genotypes only, and lacks a theoretical justification within the framework considered here. To summarise, the allele frequencies in **G**(**m**^*o*^) and the adjustments necessary for the compatibility of **G**(**m**^*o*^) and **A**_11_ should be in principle derived based on both observed marker genotypes and observed phenotypes.

Furthermore, when computing BLUP breeding values $\hat{a}$ with plugged-in parameter estimates, the uncertainty in these parameters estimates is ignored, which is an important issue in the case of the high-dimensional parameter ***ρ***. Alternatively, uncertainty in parameter estimates may be incorporated into the predictions by using a Bayesian approach. Demonstration that a Bayesian approach with prior distributions on ***ρ*** and **v** results in an method in which the pedigree-based relationship matrix is adjusted to a marker-based relationship matrix, is presented below.

### Adjusting the pedigree-based relationship matrix

The derivation of the proposed adjustment of the pedigree-based relationship matrix is based on assigning priors on the high-dimensional parameters ***ρ*** and **v** in the previously described single-step model, and then considers the first and second order moments of the marginal distribution of **M**(integrating ***ρ*** and **v**). Appendix A shows that the resulting marker distribution satisfies

$$\text{E}[M_{\text{ij}}]=0,$$

$$\text{Cov}[M_{\text{ij}},M_{i^{\prime }j^{\prime }}]={\tilde{v}}_{jj^{\prime }}Ã{\left(\gamma \right)}_{ii^{\prime }},$$

with ${\tilde{v}}_{j}={\tilde{v}}_{\mathrm{jj}}=\tilde{s}/p$, *j* = 1, … , *p*, $\tilde{s}$ being a parameter, and ${\tilde{v}}_{jj^{\prime }}=0$ when *j* ≠ *j*^*′*^. Matrix $\tilde{A}(\gamma )$ is an additive relationship matrix that satisfies the usual recursions but with the peculiar feature that base animals in the pedigree are related and inbred. Within the population of base animals, the relationship coefficient is *γ* and the inbreeding coefficient is *γ* / 2. Table 1 contains data for a small pedigree used to derive matrix $\tilde{A}$ as shown in Table 2. The mean and variance-covariance structure of **M** shown above is of the form in [3], with scaling parameter $\tilde{s}=\underset{j}{\sum }{\tilde{v}}_{j}$, and hence the breeding values **g** have a combined relationship matrix $H_{\gamma ,\tilde{s},\omega }$, where the inverse is

(3) $$H_{\gamma ,\tilde{s},\omega }^{-1}=\left[\begin{array}{cc} G_{\gamma ,\tilde{s},\omega }^{-1}-\tilde{A}{\left(\gamma \right)}_{11}^{-1} & 0\\ \;0 & 0 \end{array}\right]+\tilde{A}{\left(\gamma \right)}^{-1},$$

**Table 1:** Example pedigree

**id**	**sire**	**dam**
1	0	0
2	0	0
3	1	2
4	1	3
5	1	2
6	5	4

**Table 2:** $\tilde{A}(\gamma )$**for the pedigree in Table**1

**id**	**1**	**2**	**3**	**4**	**5**	**6**
1	1 + *γ* / 2					
2	*γ*	1 + *γ* / 2				
3	1/2 + 3*γ* / 4	1/2 + 3*γ* / 4	1 + *γ* / 2			
4	3/4 + 5*γ* / 8	1/4 + 7*γ* / 8	3/4 + 5*γ* / 8	5/4 + 3*γ* / 8		
5	1/2 + 3*γ* / 4	1/2 + 3*γ* / 4	1/2 + 3*γ* / 4	1/2 + 3*γ* / 4	1 + *γ* / 2	
6	5/4 + 3*γ* / 8	3/8 + 13*γ* / 32	5/8 + 11*γ* / 16	7/8 + 9*γ* / 16	3/4 + 3*γ* / 16	5/4 + 3*γ* / 8

with $G_{\gamma ,\tilde{s},\omega }=(1-\omega )m^{o}{\left(m^{o}\right)}^{T}/\tilde{s}+\omega \tilde{A}{\left(\gamma \right)}_{11}$. This construction is a single-step method for which the individuals in the base population are related and inbred, and the marker-based relationship matrix, $m^{o}{\left(m^{o}\right)}^{T}/\tilde{s}$, has allele frequencies equal to 0.5.

Based on the derivation above (see also Appendix A), the full marginal log-likelihood function for parameter inference becomes

(4) $$\begin{array}{lll} & \tilde{\ell }(\sigma_{a}^{2},\sigma_{e}^{2},\omega ,\gamma ,\tilde{s})\; & \;\\ & ={\tilde{\ell }}_{y|m^{o}}(\sigma_{a}^{2},\sigma_{e}^{2},\omega ,\gamma ,\tilde{s})+{\tilde{\ell }}_{\text{mark}}(\gamma ,\tilde{s}),\; \end{array}$$

where ${\tilde{\ell }}_{y|m^{o}}(\sigma_{a}^{2},\sigma_{e}^{2},\omega ,\gamma ,\tilde{s})$ for fixed *ω*, *γ* and $\tilde{s}$ is the single-step REML-log-likelihood for the phenotypes conditional on the observed marker genotypes, with marker-based relationship matrix $m^{o}{\left(m^{o}\right)}^{T}/\tilde{s}$ and pedigree-based relationship matrix $\tilde{A}(\gamma )$, and

(5) $$\begin{array}{lll} & {\tilde{\ell }}_{\text{mark}}(\gamma ,\tilde{s})=\text{const}-(pn_{1}/2)\text{log}(\tilde{s})\; & \;\\ & -(p/2)\text{log}(\det(\tilde{A}{\left(\gamma \right)}_{11}))\; & \;\\ & -\frac{p}{2\tilde{s}}\underset{ii^{\prime }}{\sum }{\left(\tilde{A}{\left(\gamma \right)}_{11}^{-1}\right)}_{ii^{\prime }}{\left(m^{o}{\left(m^{o}\right)}^{T}\right)}_{ii^{\prime }}\; \end{array}$$

is the log-likelihood of the observed marker genotypes. Using the log-likelihood in equation (4) instead of the log-likelihood in equation (2) makes the estimation of parameters feasible in practice by numeric maximization methods since the high-dimensional parameters ***ρ*** and **v** are replaced by two parameters, a parameter *γ* that determines the relationship and inbreeding of individuals in the base population and a parameter $\tilde{s}$ that scales the marker-based relationship matrix.

The computations require algorithms that compute **A**(*γ*)^−1^ and **A**_11_(*γ*) efficiently. Computations of inbreeding coefficients with related base animals have been considered by [10,11] in the context of incomplete pedigrees. In Appendix B, algorithms are presented that extend the approaches of Quaas [12] for computing **A**^−1^ and of Colleau [13] for computing **A**_11_ to the case where base animals are related and inbred.

Maximisation of the full log-likelihood in equation (4) is done by first specifying a discrete three-dimensional grid of values for parameters $\omega ,\gamma ,\tilde{s}$ and then, for each value of $\left(\omega ,\gamma ,\tilde{s}\right)$, computing the maximum values of the log-likelihood ${\tilde{\ell }}_{y|m^{o}}$ and the log-likelihood of observed marker genotypes ${\tilde{\ell }}_{\text{mark}}$. This provides a three-dimensional profile log-likelihood $\hat{\tilde{\ell }}(\omega ,\gamma ,\tilde{s})$, which can then be assessed to find the maximum.

For faster computing, an alternative to using the full log-likelihood is to determine parameters *γ* and $\tilde{s}$ based on observed marker genotypes only, i.e. by maximising ${\tilde{\ell }}_{\text{mark}}(\gamma ,\tilde{s})$, and to estimate the remaining parameter based on ${\tilde{\ell }}_{y|m^{o}}(\sigma_{a}^{2},\sigma_{e}^{2},\omega ,\gamma ,\tilde{s})$ for a grid of values for *ω*, with estimates of *γ* and $\tilde{s}$ plugged in. Setting the derivative of ${\tilde{\ell }}_{\text{mark}}(\gamma ,\tilde{s})$ with respect to $\tilde{s}$ equal to zero gives

$$0=-\frac{pn_{1}}{2\tilde{s}}+\frac{p}{2{\tilde{s}}^{2}}\underset{ii^{\prime }}{\sum }{\left(\tilde{A}{\left(\gamma \right)}_{11}^{-1}\right)}_{ii^{\prime }}{\left(m^{o}{\left(m^{o}\right)}^{T}\right)}_{ii^{\prime }},$$

which has the solution

$$\hat{\tilde{s}}\left(\gamma \right)=\underset{ii^{\prime }}{\sum }{\left(\tilde{A}{\left(\gamma \right)}_{11}^{-1}\right)}_{ii^{\prime }}{\left(m^{o}{\left(m^{o}\right)}^{T}\right)}_{ii^{\prime }}/n_{1}.$$

Substituting $\hat{\tilde{s}}(\gamma )$ into equation (5), we obtain

$$\begin{array}{lll} & {\hat{\tilde{\ell }}}_{\text{mark}}(\gamma )=\text{const}-(pn_{1}/2)\text{log}(\hat{\tilde{s}}(\gamma ))\; & \;\\ & -(p/2)\text{log}(\det(\tilde{A}{\left(\gamma \right)}_{11})),\; & \; \end{array}$$

which has to be maximised numerically to estimate *γ*.

### Adjusting the marker-based relationship matrix (G-adjust)

Alternatively, an adjustment of the form **G**_*a*_ = **G***β* + *α* is used, where **G** is the marker-based relationship matrix with allele frequencies $\hat{\rho }$ equal to the observed ones and scaling parameter $ŝ=\underset{j}{\sum }2{\hat{\rho }}_{j}(1-{\hat{\rho }}_{j})$, and parameters *α* and *β* are determined by fitting **G**_*a*_ to **A**_11_. This adjustment was used by VanRaden [14], Christensen et al. [6] and Gao et al. [15], with the first paper suggesting that *α* and *β* should be estimated by least square estimation, i.e. by minimizing the sum of squares of **G***β* + *α* − **A**_11_ and the other two papers suggesting that they should be to estimated by equating means of diagonal elements and all elements in the two matrices. Here, the later is applied and *α* and *β* are estimated by solving the two equations

(6) $$\bar{G}\beta +\alpha ={Ā}_{11}\;\text{and}\;\mathrm{dG\beta}+\alpha =dA_{11},$$

for *α* and *β*, where $\bar{G}$ and ${Ā}_{11}$ denote means of all elements in the two matrices, and *dG* and *dA* denote means of diagonal elements in the two matrices.

### Simulated example 1

This example is deliberately simple and very extreme, and constructed for the purpose of showing that parameter estimates of *γ* and $\tilde{s}$ can depend on the observed phenotypes.

The base population consists of two individuals, one sire and one dam with 15 bi-allelic markers, and their genotypes are simulated assuming independence between markers and with equal allele frequencies. Furthermore, it is assumed that the markers are independently inherited, are all QTL with allele substitution effect equal to 1, and heritability of the phenotype is equal to 1.

The two base individuals produce 100 offspring (generation 1) that all have observed phenotypes. The two individuals in generation 1 with the largest own phenotype value are selected as parents and produce 100 offspring (generation 2) that are all genotyped.

Two different approaches to estimate parameters are compared. In the first approach, all parameters are estimated using the full log-likelihood in equation (4). In the second approach, *γ* and $\tilde{s}$ are estimated based only on the log-likelihood of the observed marker genotypes in equation (5), and the remaining parameters are estimated based on the REML-log-likelihood ${\tilde{\ell }}_{y|m^{o}}(\sigma_{a}^{2},\sigma_{e}^{2},\omega ,\gamma ,\tilde{s})$, with estimates of *γ*and $\tilde{s}$ plugged-in.

### Simulated example 2

This example is inspired by a pig nucleus breeding scheme and consists of five generations in which all animals have recorded phenotypes. In each generation, 150 boars are mated to 1 500 sows to produce 15 000 offspring (50/50 males/females). For the next generation, boars with a high own phenotype value are chosen and sows are selected at random. The last three generations of selected boars are genotyped and a sixth generation of 300 candidate boars are also genotyped. The breeding value is the sum of 500 independent QTL effects simulated from a Gamma(5.4,0.42) distribution, and the heritability of the phenotype is 0.22. This dataset is described in more detail in Christensen and Lund [3].

Three different approaches are compared. In the first approach, all parameters are estimated using the full log-likelihood in equation (4). In the second approach, parameters *γ* and $\tilde{s}$ are estimated based only on the log-likelihood of the observed marker genotypes in equation (5), and the remaining parameters are estimated based on the REML-log-likelihood ${\tilde{\ell }}_{y|m^{o}}(\sigma_{a}^{2},\sigma_{e}^{2},\omega ,\gamma ,\tilde{s})$, with estimates of *γ* and $\tilde{s}$ plugged in. Finally, the G-adjust approach is used for which *α* and *β* are estimated using equation (6) and the remaining parameters are estimated by $\left(\ell_{y|m^{o}}\right)$. For all three approaches, the correlation between predicted breeding value $\hat{a}$ and true breeding value **a** for the candidate boars is reported as well as the estimated regression coefficient (reg) for the regression of **a** on $\hat{a}$, where deviation from one indicates bias.

## Results

### Simulated example 1

Table 3 shows that both *γ*and $\tilde{s}$ were smaller when estimated with the full log-likelihood than with the log-likelihood of the observed marker genotypes.

**Table 3:** Parameter estimates obtained with simulated dataset 1

	${\tilde{\ell }}_{y|m^{o}}+{\tilde{\ell }}_{\text{mark}}$	${\tilde{\ell }}_{\text{mark}}$**&**${\tilde{\ell }}_{y|m^{o}}$
$\hat{\gamma }$	0.524	0.542
$\hat{\tilde{s}}/(p/2)$	1.068	1.081
$\hat{\omega }$	0.005	0.005
${\hat{\sigma }}_{a}$	1.186	1.355
${\hat{\sigma }}_{e}$	2.370	2.312

Parameters were estimated either, jointly using the full log-likelihood $\tilde{\ell }={\tilde{\ell }}_{y|m^{o}}+{\tilde{\ell }}_{\text{mark}}$, or by first estimating *γ* and $\tilde{s}$ using ${\tilde{\ell }}_{\text{mark}}$ and then the other parameters using ${\tilde{\ell }}_{y|m^{o}}$.

### Simulated example 2

**Figure 1:**
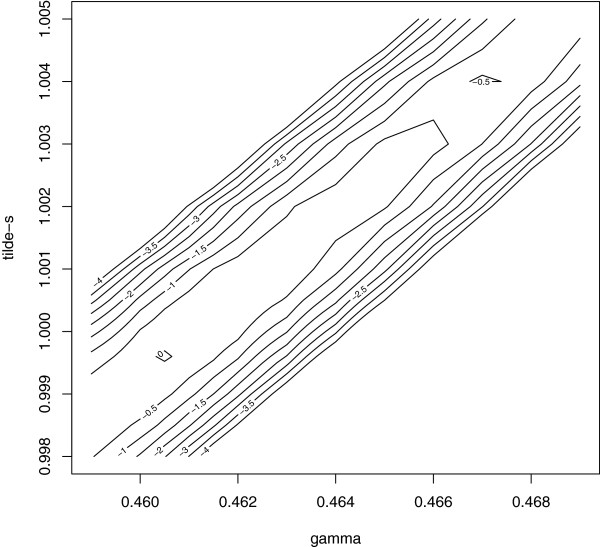
**Profile log-likelihood for *γ *and **$\tilde{s}$**.** A contour-plot of the profile log-likelihood for parameters *γ* and $\tilde{s}$ based on the full log-likelihood in equation (5); the plot is constructed with values in a discrete grid (explaining the roughness of the plot) that have been standardised such that the maximum value is zero.

**Figure 2:**
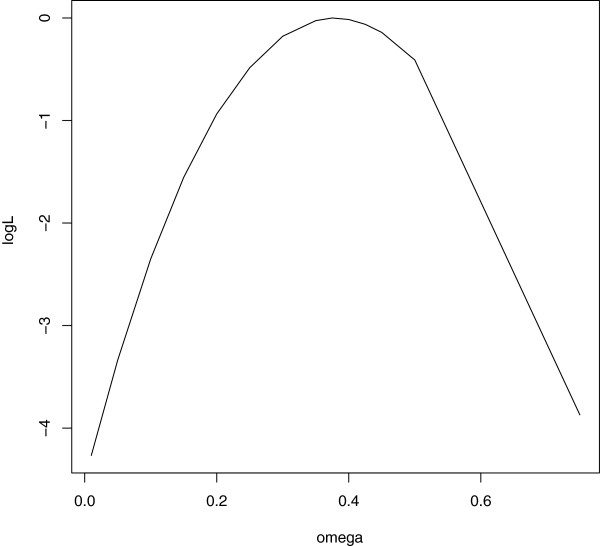
**Profile log-likelihood for *ω***. The profile log-likelihood function for parameter *ω* based on the full log-likelihood in equation (5); the plot is constructed with values in a discrete grid that have been standardised such that the maximum value is zero.

Table 4 shows that *γ* and $\tilde{s}$ were slightly smaller when estimated with the full log-likelihood than with the log-likelihood of the observed marker genotypes. Parameter *ω* was about 0.375 whether estimated with the full log-likelihood or with the log-likelihood ${\tilde{\ell }}_{y|m^{o}}$, with parameter estimates of *γ* and $\tilde{s}$ from ${\tilde{\ell }}_{\text{mark}}$ plugged-in. When presented in three dimensions, the profile log-likelihood showed a very weak association between $\left(\gamma ,\tilde{s}\right)$ and *ω*. A contour plot of the profile log-likelihood surface for *γ* and $\tilde{s}$ is shown in Figure 1 and the profile log-likelihood function for *ω* is shown in Figure 2.

**Table 4:** Parameter estimates and prediction performance obtained with simulated dataset 2

	${\tilde{\ell }}_{y|m^{o}}+{\tilde{\ell }}_{\text{mark}}$	${\tilde{\ell }}_{\text{mark}}$**&**${\tilde{\ell }}_{y|m^{o}}$	***G*-adjust**
$\hat{\gamma }$	0.4605	0.4615	
$\hat{\tilde{s}}/(p/2)$	0.9996	1.0003	
*α*			0.0134
*β*			1.0074
$\hat{\omega }$	0.375	0.375	0.60
${\hat{\sigma }}_{a}$	6.507	6.512	5.084
${\hat{\sigma }}_{e}$	15.816	15.816	15.797
$\text{Cor}(\hat{a},a)$	0.536	0.536	0.493
reg	1.17	1.17	1.34

Parameters were estimated either, jointly using the full log-likelihood $\tilde{\ell }={\tilde{\ell }}_{y|m^{o}}+{\tilde{\ell }}_{\text{mark}}$, or by first estimating *γ* and $\tilde{s}$ using ${\tilde{\ell }}_{\text{mark}}$ and then the other parameters using ${\tilde{\ell }}_{y|m^{o}}$; the last column contains parameter values estimated with the single-step method with an adjusted marker-based relationship matrix, in which first *α* and *β* were estimated using equation (6) and then the remaining parameters were estimated using $\left(\ell_{y|m^{o}}\right)$; the last two rows show the correlation between predicted and true breeding values, $\text{Cor}(\hat{a},a)$, for candidate boars, which is a measure of prediction performance, and the regression coefficient (reg) for the regression of **a**on $\hat{a}$.

Estimated parameters obtained with the G-adjust approach are also shown in Table 4, where it should be noted that $\hat{\omega }=0.6$.

In terms of prediction performance, the correlation between predicted and true breeding value and the regression coefficient were 0.536 and 1.17, respectively, in both cases when the pedigree-based matrix was adjusted, but 0.493 and 1.34, respectively, when the G-adjust approach was used.

## Discussion

This paper provides a coherent approach to handle the issue of compatibility between pedigree-based and marker-based relationship matrices in single-step genetic evaluation. The approach is computationally fast and feasible for large datasets, as is the case for previously developed single-step methods. Parameter *γ* can adjust the pedigree-based relationship matrix to the marker-based relationship matrix that is scaled by parameter $\tilde{s}$, and these two parameters should in principle be estimated using the full log-likelihood for observed phenotypes and observed marker genotypes. This is computationally feasible by computing the full log-likelihood values for parameters *γ*, $\tilde{s}$ and *ω* in a three-dimensional grid. However, in practice this can be computationally burdensome and an appealing alternative is to estimate *γ* and $\tilde{s}$ based on the observed marker genotypes only. Analysis of simulated datasets, shows that the estimates of parameters *γ* and $\tilde{s}$ depend on the observed phenotypes as well as on the observed marker genotypes, although this dependence is not too large. A conjecture is that in a scenario with a small number of genotyped animals it is more important to use the full log-likelihood than in a scenario with a large number of genotyped animals. Based on these two simulated datasets only, no general conclusion can be made and further studies are needed to determine in which scenarios it would be safe to base inference on a two-step procedure in which *γ* and $\tilde{s}$ are estimated based on the observed marker genotypes only, and the remaining parameters are then estimated by the log-likelihood of the phenotypes conditional on the observed marker genotypes.

Using the approach developed in this paper may also provide insight on performance of other approaches for making marker-based and pedigree-based relationship matrices compatible in single-step genetic evaluation. Examples of such approaches are the adjustments of the marker-based relationship matrix reported in [4-6,14,15] and also investigated here, and the approach by Meuwissen et al. [16]. In [16], first, an average of position specific identical by descent matrices based on linkage information [17] (hereafter denoted **G**_*FG*_) were used instead of the combined relationship matrix (1), and second, the final method consisted of replacing the pedigree-based relationship matrix **A** by the matrix **G**_*FG*_ in matrix (1) and then adjusting the marker-based relationship matrix to the **G**_*FG*_ matrix. Based on simple simulated datasets, without selection, the method resulted in high accuracy and low bias. The computation of **G**_*FG*_ is computationally burdensome and therefore this approach was not considered here. It should be noted that for these other approaches, the combined relationship matrix is constructed based on observed marker genotypes only, and the inference on remaining parameters is based on the log-likelihood conditional on the observed marker genotypes. The approach in this paper may provide guidelines on when it is safe to base inference on such procedures in which, first the observed marker genotypes are used to construct a combined relationship matrix, and second the remaining inference is based on the log-likelihood for the phenotypes conditional on observed marker genotypes.

Evaluation of accuracy and bias of prediction with the simulated dataset 2 shows that the approaches based on adjusting the pedigree-based relationship matrix, where $\text{Cor}(\hat{a},a)=0.536$ and reg = 1.17, seem to perform better than the G-adjust approach, where $\text{Cor}(\hat{a},a)=0.493$ and reg = 1.34. However, $\hat{\omega }$ is also larger in the second case (0.60) than in the first case (0.375). If *ω* is set at 0.375 in the second case then $\text{Cor}(\hat{a},a)=0.534$, which is close to the value in the first case, but reg is somewhat larger, i.e. 1.30. If *ω* is set at 0.1, then $\text{Cor}(\hat{a},a)=0.545$ and reg = 1.00 in the first case, and $\text{Cor}(\hat{a},a)=0.550$ and reg = 1.12 in the second case, and if *ω* is set at 0.01, then $\text{Cor}(\hat{a},a)=0.548$ and reg = 1.05 in the second case. Thus, the first two approaches seem to perform better in terms of estimating a more proper *ω* and have somewhat less bias, while accuracy is similar for all three approaches.

This paper suggests that the pedigree-based relationship matrix should be adjusted instead of the marker-based relationship matrix. On the one hand, from a practical point of view this would be rather inconvenient since standard software used for REML estimation and BLUP would have to be modified, but on the other hand, it is conceptually simpler and may be easier to extend. For example, when the population consists of a mixture of breeds, it may be simpler to extend the approach in this paper and specify a parametric structure on the relationships of the animals in the base population and estimate those parameters, instead of developing an appropriate way of adjusting the marker-based relationship matrix of the genotyped animals across breeds. An additional issue is the interpretation of the genetic variance parameter $\sigma_{a}^{2}$. The parameter estimates in Table 1 are ${\hat{\sigma }}_{a}^{2}=6.507$ when $\hat{\gamma }=0.4605$, ${\hat{\sigma }}_{a}^{2}=6.512$ when $\hat{\gamma }=0.4615$, and ${\hat{\sigma }}_{a}^{2}=5.084$ for G-adjust, where base animals are unrelated, i.e. *γ* = 0. This may seem counter-intuitive since the variance of breeding values for a base animal is $\sigma_{a}^{2}(1+\gamma /2)$. However, when studying the inverse relationship matrix $\tilde{A}{\left(\gamma \right)}^{-1}$, then the averages of diagonal elements are 3.807, 3.809 and 2.93 for the three cases, respectively, and since 3.807/6.507, 3.809/6.512 and 2.93/5.084 are roughly the same, the parameter estimates actually make good sense, although the interpretation of $\sigma_{a}^{2}$ is unclear. Finally, it should be noted that parameter *γ* could influence to some extent accuracy and bias of prediction, and neither *γ* = 0 nor *γ* estimated to make marker-based and pedigree-based relationships compatible, would be optimal for that purpose. Adjusting the pedigree-based relationship instead of the marked-based relationship matrix to make the two matrices compatible in a single-step method is an interesting alternative.

For the simulated dataset 1, the estimate of the relative polygenic weight *ω* was about 0, as was expected since in the simulation the markers were assumed to capture all the genetic variation. However, for the simulated dataset 2, $\hat{\omega }$ was large when adjusting the pedigree-based relationship matrix, i.e. 0.375, and even larger, i.e. 0.60, when the G-adjust approach was used. For the second dataset, the prediction biases were reduced when *ω* was set at 0.1. This poses the question whether it is actually possible to estimate *ω* at a reasonable value from data, or whether *ω* should be determined manually to control the bias. It should be noted that confidence intervals of this parameter are large in these examples and further studies are needed.

## Conclusions

Using the full log-likelihood and adjusting the pedigree-based relationship matrix to be compatible with the marker-based relationship matrix provides a new and interesting approach to handle the issue of compatibility between the two matrices in single-step genetic evaluation.

## Appendix A

The derivation of the proposed adjustment of the pedigree-based relationship matrix is based on assigning priors on the high-dimensional parameters ***ρ*** and **v** in the previously described single-step model. This derivation is presented here.

The model for the marker genotypes, **M**, given the allele frequencies ***ρ*** and variances **v**, is as in [3],

$$\text{E}[M_{\text{ij}}|\rho_{j}]=2\rho_{j}-1,\;\;\text{Cov}[M_{\text{ij}},M_{i^{\prime }j}|v_{j}]=v_{j}A_{ii^{\prime }},$$

$$\text{Cov}[M_{\text{ij}},M_{i^{\prime }j^{\prime }}|v_{jj^{\prime }}]=v_{jj^{\prime }}A_{ii^{\prime }},$$

where *v*_*j*_ = *v*_*j*,*j*_ is a parameter and $v_{jj^{\prime }}=0$ for *j* ≠ *j*^*′*^. Prior distributions for ***ρ ***and **v** are not specified explicitly, only the necessary assumptions are stated. Assuming that the alleles are randomly labeled 1 and 2, then a priori the expected allele frequency is E[*ρ*_*j*_] = 0.5. Additional assumptions are that *η*_1_ = Var[*ρ*_*j*_] and *η*_2_ = E[*v*_*j*_] do not depend on *j*, and that $\text{Cov}[\rho_{j},\rho_{j^{\prime }}]=0$ for *j* ≠ *j*^*′*^.

With these prior distributions, a marginal distribution of **M** may be obtained by integrating ***ρ*** and **v**. Using well-known formulas for conditional expectations and covariances, the mean and covariances become

$$\text{E}[M_{\text{ij}}]=\text{E}[\text{E}[M_{\text{ij}}|\rho_{j}]]=\text{E}[2\rho_{j}-1]=0,$$

$$\begin{array}{lll} & \text{Cov}[M_{\text{ij}},M_{i^{\prime }j}]\; & \;\\ & =\text{E}[\text{Cov}[M_{\text{ij}},M_{i^{\prime }j}|\rho_{j},v_{j}]]\; & \;\\ & \;+\text{Cov}[\text{E}[M_{\text{ij}}|\rho_{j},v_{j}],\text{E}[M_{i^{\prime }j}|\rho_{j},v_{j}]]\; & \;\\ & =\text{E}[v_{j}A_{ii^{\prime }}]+\text{Var}(2\rho_{j}-1)=\eta_{2}A_{ii^{\prime }}+4\eta_{1},\; & \; \end{array}$$

$$\begin{array}{lll} & \text{Cov}[M_{\mathrm{ij}},M_{i^{\prime }j^{\prime }}]\; & \;\\ & =\text{E}[\text{Cov}[M_{\mathrm{ij}},M_{i^{\prime }j^{\prime }}|\rho ,v]]\; & \;\\ & \;+\text{Cov}[\text{E}[M_{\mathrm{ij}}|\rho ,v],\text{E}[M_{i^{\prime }j^{\prime }}|\rho ,v]]\; & \;\\ & =\text{E}[0]+4\text{Cov}[\rho_{j},\rho_{j^{\prime }}]=0\;\text{for}\;j\neq j^{\prime }.\; & \; \end{array}$$

Defining ${\tilde{v}}_{j}=2\eta_{1}+\eta_{2}$, and *γ* = 4*η*_1_ / (2*η*_1_ + *η*_2_), we obtain

$$\text{Cov}[M_{\mathrm{ij}},M_{i^{\prime }j}]=Ã{\left(\gamma \right)}_{ii^{\prime }}{\tilde{v}}_{j},$$

with $Ã{\left(\gamma \right)}_{ii^{\prime }}=(1+\gamma /2)A_{ii^{\prime }}+\gamma$. It is not difficult to see that $\tilde{A}(\gamma )$ satisfies the usual recursions for an additive relationship matrix. Requiring that 0 ≤ *γ* < 1 is equivalent to making a further assumption that 2*η*_1_ < *η*_2_ (this is for example satisfied when *ρ*_*j*_∼*U*] 0;1 and [*v*_*j*_ = 2*ρ*_*j*_(1 − *ρ*_*j*_), where *η*_1_ = 1/12 and *η*_2_ = 1/3). The first and second order moments of **M** are therefore of the form considered in [3], with pedigree-based relationship matrix $\tilde{A}(\gamma )$, allele frequencies ${\tilde{\rho }}_{j}=0.5$, scaling parameter $\tilde{s}=\underset{j}{\sum }{\tilde{v}}_{j}$ with ${\tilde{v}}_{j}$ not depending on *j*, and marker-based relationship matrix $m^{o}{\left(m^{o}\right)}^{T}/\tilde{s}$.

Note that the random labelling of alleles as 1 or 2 is not important because the resulting expression does not depend on how alleles are labelled.

Using ${\tilde{v}}_{j}=\tilde{s}/p$, the log-likelihood for the observed marker genotypes becomes

$$\begin{array}{lll} & {\tilde{\ell }}_{\text{mark}}(\gamma ,\tilde{s})=\text{const}-(pn_{1}/2)\text{log}(\tilde{s})\; & \;\\ & -(p/2)\text{log}(\det(\tilde{A}{\left(\gamma \right)}_{11}))\; & \;\\ & -(1/2)\underset{ii^{\prime }}{\sum }{\left({\left(\tilde{s}{\tilde{A}}_{11}(\gamma )/p\right)}^{-1}\right)}_{ii^{\prime }}{\left(m^{o}{\left(m^{o}\right)}^{T}\right)}_{ii^{\prime }}.\; & \; \end{array}$$

This log-likelihood may also be viewed as the log-likelihood for $\left(M^{o}{\left(M^{o}\right)}^{T}\right)$ being Wishart distributed $W_{n_{1}}(\tilde{s}{\tilde{A}}_{11}(\gamma )/p,p)$.

## Appendix B

The aim here is to present algorithms for computing ${\left(\tilde{A}(\gamma )\right)}^{-1}$ and $\tilde{A}{\left(\gamma \right)}_{11}$ by recursions. Computations of inbreeding coefficients with related based animals were considered by [10,11]. For simplicity, let’s assume that, either both parents are known or both parents are unknown. This is not an important restriction in practice, since the case with one unknown parent can be handled by assigning an artificial animal-id to this unknown parent and by letting both its parents be unknown.

Let us partition matrix $\tilde{A}$ (skipping the dependence on *γ* in the notation from now and onwards) according to whether the animals are base animals (both parents unknown) or not

$$\tilde{A}=\left[\begin{array}{cc} {\tilde{A}}^{\text{bas}}\; & \;{\tilde{A}}^{\text{bas},\text{nonbas}}\\ {\tilde{A}}^{\text{nonbas},\text{bas}} & \;\;\;\;{\tilde{A}}^{\text{nonbas}} \end{array},\;\right].$$

Similar to the usual **A** matrix (see [18]) a decomposition exists

$$\tilde{A}=T\;\left[\begin{array}{cc} {\tilde{A}}^{\text{bas}} & \;0\\ \;0\;\;\;\; & \;\tilde{D} \end{array},\;\right]\;T^{T},$$

where **T** is a lower triangular matrix with entries *T*_*ii*_ = 1, *T*_*ik*_ = (*T*_*f*(*i*)*k*_ + *T*_*m*(*i*)*k*_) / 2 when *i* has known parents *f*(*i*) and *m*(*i*) and *i* > *k*, and *T*_*ik*_ = 0 otherwise (*k* > *i* or *i* has unknown parents), and $\tilde{D}$ is a diagonal matrix containing the variance of the Mendelian sampling part for matrix ${\tilde{A}}^{\text{nonbas}}$, i.e. ${\tilde{D}}_{\mathrm{ii}}=1-({Ã}_{f(i)f(i)}+{Ã}_{m(i)m(i)})/4$. Comparing this decomposition to the decomposition for the usual **A** matrix, then matrix ${\tilde{A}}^{\text{bas}}$ has replaced an identity matrix and $\tilde{D}$ has replaced a diagonal matrix containing the Mendelian sampling terms related to **A** for the animals with known parents.

The inverse matrix becomes

$${\tilde{A}}^{-1}={\left(T^{-1}\right)}^{T}\left[\begin{array}{cc} {\left({\tilde{A}}^{\text{bas}}\right)}^{-1} & \;\;\;\;0\\ \;0\;\;\;\;\; & \;{\tilde{D}}^{-1} \end{array},\;\right]T^{-1}.$$

Here **T**^−1^ is a lower triangular matrix with ones on the diagonal and the only non-zero elements being −1 / 2 for offspring-parent entries, and matrix ${\left({\tilde{A}}^{\text{bas}}\right)}^{-1}$ has diagonal elements equal to (1 + (*n*_*bas*_−3/2)*γ*) / ((1−*γ*/2)(1 + (*n*_*bas*_−1/2)*γ*)) and off-diagonal elements equal to − *γ* / ((1 − *γ* / 2)(1 + (*n*_*bas*_ − 1 / 2)*γ*)), where *n*_*bas*_ is the dimension of ${\tilde{A}}^{\text{bas}}$.

A procedure to obtain the diagonal of $\tilde{A}$ and the inverse ${\left(\tilde{A}\right)}^{-1}$ can be constructed similar to the algorithm by Quaas [12], which utilises the form $\tilde{A}=LL^{T}$ where

$$L=T\;\left[\begin{array}{cc} \sqrt{{\tilde{A}}^{\text{bas}}} & \;\;\;\;0\\ 0\; & \;\sqrt{\tilde{D}} \end{array},\;\right].$$

Matrix $\sqrt{{\tilde{A}}_{\text{bas}}}$ is a lower triangular matrix such that ${\tilde{A}}_{\text{bas}}=\sqrt{{\tilde{A}}_{\text{bas}}}{\left(\sqrt{{\tilde{A}}_{\text{bas}}}\right)}^{T}$. First, matrix $\sqrt{{\tilde{A}}_{\text{bas}}}$ is computed and ${Ã}_{\mathrm{ii}}$ is set to 1 + *γ* / 2 for the base animals. Second, the remaining part of the algorithm is the same as in Quaas [12], where recursively, rows in *L* are computed using ${\tilde{D}}_{\mathrm{ii}}=1-({Ã}_{f(i)f(i)}+{Ã}_{m(i)m(i)})/4$ and ${Ã}_{\mathrm{ii}}=\overset{i}{\underset{k=1}{\sum }}L_{\mathrm{ik}}^{2}$. Obtaining ${\left(\tilde{A}\right)}^{-1}$ at the same time is done by first setting elements in ${\left(\tilde{A}\right)}^{-1}$ for base animals equal to the elements in ${\left({\tilde{A}}^{\text{bas}}\right)}^{-1}$, and then adding elements to ${\left(\tilde{A}\right)}^{-1}$ in the usual way (assuming a half-stored format, add $1/{\tilde{D}}_{\mathrm{ii}}$ to the (*i*,*i*) element, add $-1/(2{\tilde{D}}_{\mathrm{ii}})$ to the (*i*,*f*(*i*)) and (*i*,*m*(*i*)) elements, and add $1/(4{\tilde{D}}_{\mathrm{ii}})$ to the (*f*(*i*),*f*(*i*)), (*m*(*i*),*m*(*i*)) and (*m*(*i*),*f*(*i*)) elements).

Matrix ${\tilde{A}}_{11}$ can be obtained by a modification of the Colleau algorithm [13,19,20] by using an algorithm to compute $\tilde{A}x$, where **x** is a vector. The product of matrix $\tilde{A}$ and vector **x** is

(7) $$\tilde{A}x=T\;\left[\begin{array}{cc} {\tilde{A}}^{\text{bas}} & \;0\\ \;0\;\;\;\; & \;\tilde{D} \end{array},\;\right]T^{T}x.$$

Therefore, an algorithm (with notation similar to that in Appendix A in Misztal et al. [19]) consists of first computing **r** = **T**^T^**x** by solving the sparse system $\left({\left(T^{-1}\right)}^{T}r=x\right)$ for **r**, then computing

$$t=\left[\begin{array}{cc} {\tilde{A}}^{\text{bas}} & \;0\\ \;0\;\;\; & \;\tilde{D} \end{array},\;\right]\;r=\left[\begin{array}{c} (1-\gamma /2)r^{\text{bas}}+\gamma 1^{T}r^{\text{bas}}1\\ \;\;\tilde{D}r^{\text{nonbas}} \end{array}\right],$$

and finally computing $\tilde{A}x=T^{T}t$ by solving the sparse system ${\left(T^{-1}\right)}^{T}(\tilde{A}x)=r$ for $\tilde{A}x$.

## Competing interests

The author declares that he has no competing interests.

## Authors’ contributions

The author has read and approved the final manuscript.
