# Correction: Compatibility of pedigree-based and marker-based relationship matrices for single-step genetic evaluation

**DOI:** 10.1186/1297-9686-46-20

**Published:** 2014-04-23

**Authors:** Ole F Christensen

**Affiliations:** 1 Department of Molecular Biology and Genetics, Center for Quantitative Genetics and Genomics, Aarhus University, Blichers Alle’ 20, P.O. BOX 50DK-8830 Tjele, Denmark

## Correction

In our publication [1], a confusing sign error exists in Appendix A, page 9, first column, line 18, where it incorrectly says that matrix **A**(*γ*) is defined as $A{\left(\gamma \right)}_{ii^{\prime }}=\left(1+\gamma /2\right)A_{ii^{\prime }}+\gamma$.

Instead it should have said that it is defined as $A{\left(\gamma \right)}_{ii^{\prime }}=\left(1-\gamma /2\right)A_{ii^{\prime }}+\gamma$. Defined in this way, matrix **A**(*γ*) is an additive relationship matrix that satisfies the usual recursions but has the peculiar feature that base animals in the pedigree are related (with relationship coefficient *γ*) and inbreed (with inbreeding coefficient *γ*/2) as mentioned in the main text of [1].

I apologise for any inconvenience.
